# The Regulator of G Protein Signaling Homologous Domain of G Protein-Coupled Receptor Kinase 2 Mediates Short-Term Desensitization of β3-Adrenergic Receptor

**DOI:** 10.3389/fphar.2020.00113

**Published:** 2020-02-21

**Authors:** Emiliana Echeverría, Maia Cabrera, Valeria Burghi, Máximo Sosa, Sonia Ripoll, Agustín Yaneff, Federico Monczor, Carlos Davio, Carina Shayo, Natalia Fernández

**Affiliations:** ^1^ Facultad de Farmacia y Bioquímica, Universidad de Buenos Aires, Buenos Aires, Argentina; ^2^ Facultad de Farmacia y Bioquímica, Instituto de Investigaciones Farmacológicas (ININFA, UBA, CONICET), Universidad de Buenos Aires, Buenos Aires, Argentina; ^3^ Laboratorio de Patología y Farmacología Molecular, Instituto de Biología y Medicina Experimental (IByME), CONICET, Buenos Aires, Argentina

**Keywords:** desensitization, GRK2, RGS, β3AR, cardiomyocytes

## Abstract

G protein coupled receptor (GPCR) kinases (GRKs) are key regulators of GPCR signaling. Canonical mechanism of GPCR desensitization involves receptor phosphorylation by GRKs followed by arrestin recruitment and uncoupling from heterotrimeric G protein. Although β3-adrenergic receptor (β3AR) lacks phosphorylation sites by GRKs, agonist treatment proved to induce β3AR desensitization in many cell types. Here we show that GRK2 mediates short-term desensitization of β3AR by a phosphorylation independent mechanism but mediated by its domain homologous to the regulator of G protein signaling (RGS). HEK293T cells overexpressing human β3AR presented a short-term desensitization of cAMP response stimulated by the β3AR agonist, BRL37344, and not by forskolin. We found that β3AR desensitization was higher in cells co-transfected with GRK2. Similarly, overexpression of the RGS homology domain but not kinase domain of GRK2 increased β3AR desensitization. Consistently, stimulation of β3AR increased interaction between GRK2 and Gαs subunit. Furthermore, in rat cardiomyocytes endogenously expressing β3AR, transfection with dominant negative mutant of RH domain of GRK2 (GRK2/D110A) increased cAMP response to BRL37344 and inhibited receptor desensitization. We expect our study to be a starting point for more sophisticated characterization of the consequences of GRK2 mediated desensitization of the β3AR in heart function and disease.

## Introduction

β3-adrenergic receptor (β3AR) was the last member of the β adrenoreceptors to be identified (Emorine et al., 1989) and presents a more restrictive expression profile than their counterparts. It can be found in tissues such as vascular and intestinal smooth muscle, urinary bladder, kidney tubules, brown fat and cardiovascular system. Although β3AR agonists are clinically used only for the treatment of overactive bladder syndrome (Sacco and Bientinesi, 2012; Igawa and Michel, 2013), β3AR has been also postulated as a putative target for the treatment of other diseases such as heart failure (Cannavo and Koch, 2017).

β3AR belongs to the G protein-coupled receptor (GPCR) family characterized by seven transmembrane domains (Emorine et al., 1989), and can signal through both Gi and Gs protein depending on the tissue and species (Kohout et al., 2001; Pott et al., 2006) and even in a same cellular context (Gerhardt et al., 1998). It shares only 40–50% amino acid sequence with other adrenergic subtypes (Granneman et al., 1993) and its gene comprises one or two introns depending on the animal species. The first exon encodes the initial 402 amino acids of the receptor, while the following encode the last six or twelve amino acids in the C-terminus of longer splice variants (Granneman et al., 1992; Granneman et al., 1993; van Spronsen et al., 1993). Its C-terminal divergence respect to the other β-AR subtypes has consequences in its pharmacologic regulation and its sensitivity to desensitization. Since β3AR lacks potential phosphorylation sites in its third intracellular loop and C-terminal tail, it has been thought to be insensitive to cAMP dependent protein kinase (PKA) or GPCR specific kinases (GRKs) mediated desensitization, particularly as compared to β1- and β2-adrenoceptors (Hausdorff et al., 1990). However, phosphorylation is not the only GRK-mediated mechanism for GPCR desensitization. Increasing evidence demonstrates that GPCRs response can be attenuated by proteins with RGS (regulator of G protein signaling) domain, acting directly on G-proteins by increasing their GTP hydrolysis rate (GAP activity) or by precluding Gα-effector interaction when binding to activated Gα subunit occurs (Evron et al., 2012). GRKs, as members of the RGS family of proteins, present an N-terminal RGS homology (RH) domain that has been involved in phosphorylation independent signaling attenuation of several GPCRs (Sallese et al., 2000; Usui et al., 2000; Reiter et al., 2001; Dhami et al., 2002; Iwata et al., 2005; Namkung et al., 2009; Fernandez et al., 2011).

Up to the present, conclusions about susceptibility of β3AR to agonist-induced desensitization diverge. Reports showed that β3AR was resistant to both short term and long term receptor desensitization in endogenous and heterologous expression systems (Granneman, 1992; Liggett et al., 1993; Nantel et al., 1993; Milano et al., 2018). Accordingly, substitution of the second and third cytoplasmic loops and C-terminal tail of the β3AR with those of β2AR restored desensitization (Jockers et al., 1996). In contrast, studies carried out in diverse experimental models have shown that agonist-induced β3AR desensitization occurs (Chaudhry and Granneman, 1994; Scarpace et al., 1999; Hutchinson et al., 2000; Vrydag et al., 2009), and that long term treatment with isoproterenol induced β3AR-mediated cAMP response desensitization in HEK293 transfected cells by reducing the activity of adenylyl cyclase (Vrydag et al., 2009; Michel-Reher and Michel, 2013; Okeke et al., 2019).

In this context, we aimed to evaluate agonist induced short-term desensitization of human β3AR expressed in HEK293 cells, and the underlying mechanism. Pre-exposure of cells to the β3AR agonist, BRL37344 led to a 40% reduction in cAMP response to the agonist and not to forskolin. This effect was not attributable to receptor endocytosis or a reduction in Gs protein levels. We found that among cardiac GRKs, GRK2 but not GRK5 diminished cAMP response of β3AR and increased receptor desensitization. Stimulation of β3AR with BRL37344 increased interaction between GRK2 and Gαs subunit. Overexpression of GRK2 kinase death mutant or RH domain also increased β3AR desensitization. Similarly, in rat cardiomyocytes that natively express β3AR, dominant negative mutant of the RH domain of GRK2 increased cAMP response to BRL37344 and attenuated desensitization.

## Materials and Methods

### Cell Culture

HEK293T (Human embryonic kidney, ATCC^®^ CRL-3216™) cells were cultured in Dulbecco’s modified Eagle’s medium (DMEM) supplemented with 10% foetal bovine serum (FBS) and 5μg/ml gentamicin at 37°C in humidified atmosphere containing 5% CO2.

Neonatal rat cardiomyocytes were isolated from the heart of 18-day-old embryos of Sprague-Dawley rats obtained from the animal facilities of the Facultad de Farmacia y Bioquímica, Universidad de Buenos Aires. Briefly, after decapitation, hearts were quickly excised, the atria were cut off, then ventricles were excised and transferred to fresh ice-cold PBS buffer and were minced with fine scissors into 1–3 mm^3^ pieces after washing blood away from the heart lumen. The minced tissue was subjected to digestion six times in a balanced salt solution containing collagenase type IV (1mg/ml). The disaggregated cells were collected by centrifugation at 400 g for 5 min and maintained in DMEM-F12 containing 10% FBS, 10μg/ml insulin, 10μg/ml holo-transferrin, 100μM bromodeoxyuridine (to prevent proliferation of non-myocyte cells), 10,000U/ml penicillin and 10mg/ml streptomycin. To reduce the contribution of non-myocardial cells, cells were pre-plated for 1 h. The myocyte-enriched cells remaining in suspension were plated at a density of 0.5×10^6^ cells/well in 12-well plates.

Using this method, we routinely obtain cultures with ≈90–95% myocytes, as assessed by microscopic observation of rod-shaped cells spontaneously beating. All animal experiments comply with the ARRIVE guidelines (Kilkenny et al., 2010) and were carried out in accordance with the guidelines of the National Institute of Health *Guide for the Care and Use of Laboratory Animals* (2011). All efforts were made to minimize the number of animals used and their suffering. This study was approved by the Animal Use and Care Committee from the Facultad de Farmacia y Bioquímica, Universidad de Buenos Aires (Res. 4662).

### Transient and Stable Transfections

For transient transfections, HEK293T and HEKT Epac-S^H187^ cells were grown to 80–90% confluency. cDNA constructs were transfected into cells using K2 Transfection System (Biontex, Munich, Germany). The transfection protocol was optimized as recommended by the supplier. Assays were always performed 48 h after transfection. The expression of the constructs was confirmed by immunoblotting using specific antibodies.

Cardiomyocytes were plated in 12-well culture plates at a density of 0.5×10^6^ cells/well in 800μl medium. The day after, cells were transfected with 1µg of cDNA or 1µg of siRNA constructs using K2 Transfection System (Biontex, Munich, Germany). Twenty-four hours after transfection, media was replaced. Assays were always performed 48 h after transfection. The efficacy of the construct was confirmed by immunoblotting using specific antibodies.

Stable HEK293T expressing pcDNA3.1/Zeo(1)-mTurquoise2-EPAC-cp173Venus-Venus (Epac-S^H187^) (HEKT-Epac-S^H187^) cells were obtained by transfection of HEK293T using K2 Transfection System (Biontex, Munich, Germany). Twenty-four hours after transfection, cells were seeded in the presence of 25µg/ml zeocine (InvivoGen) for 2 weeks, and clonal selection was carried out in 96-well plates for 2 weeks. Clones were tested for Epac-SH187 by fluorescence espectra (450–650 nm) measurements in a FlexStation 3 Multi-Mode Microplate Reader (Molecular Devices) with excitation at 430 nm. The HEKT Epac-S^H187^ clone with higher fluorescence emission was chosen for further experiments. The stable clone was grown in DMEM medium supplemented with 10% FBS, 50 μg/ml gentamicin and 12.5 μg/ml zeocin.

### Plasmid and siRNA Constructions

Histamine type 2 receptor (H2R), GRK2, and GRK5 cDNAs were previously subcloned into the pCEFL vector (pCEFL H2R, pCEFL GRK2, and pCEFL GRK5) in our laboratory (Shayo et al., 2001). pCEFLHA RHPH construct derived from GRK2 was previously obtained in our laboratory (Fernandez et al., 2011). pCDNA3.1 HA-β3-adrenergic receptor of human origin was obtained from Missouri S&T cDNA Resource Center (Rolla, MO). pcDNA3 GRK2-D110A was kindly provided by Dr. R. Sterne-Marr (Biology Dept., Siena College, Loudonville, NY). pcDNA3 GRK2-K220R and pcDNA3 GRK2-R106A/K220R were a kind gift from Dr. J. Benovic (Thomas Jefferson University, Microbiology and Immunology Department, Kimmel Cancer Center, Philadelphia). Rat β3AR siRNA (CAACAGGUUUUGAUGGCUAU) and Non-Targeting siRNA (UAAGGCUAUGAAGAGAUAC) was purchased to Horizon Discovery Ltd. (Cambridge, UK). The mTurquoise2-EPAC-cp173Venus-Venus (Epac-S^H187^) construct was provided by Dr. KeesJalink (Cell Biophysics & Imaging Group, Netherlands Cancer Institute) (Klarenbeek et al., 2015).

### Western Blot Assays

For western blot assays, cells were lysed in 50mM Tris-HCl pH 6.8, 2% SDS, 100mM 2-mercaptoethanol, 10% glycerol and 0.05% bromophenol blue and sonicated to shear DNA. Total cell lysates were resolved by SDS-PAGE. Blots were incubated with primary anti: HA, β-tubulin, Gαs/olf, GRK2, and GRK5 antibodies (Santa Cruz Biotechnology, CA; see materials for details), followed by horseradish peroxidase conjugated anti-rabbit or anti-mouse antibodies (Vector Laboratories, CA; see materials for details) and developed by enhanced chemiluminesence (ECL) following the manufacturer’s instructions (Amersham Life Science, England).

### Immunofluorescence

HEK293T cells were washed with PBS 48 h after transfection with pCDNA3.1-HA β3AR or empty vector and fixed in 2% formaldehyde in PBS for 10 min. The non-specific binding sites were blocked for 30 min with blocking solution (PBS containing 1% BSA). Then cells were incubated for 1 h at room temperature with the anti-HA antibody (1:50). Following washing with PBS, cells were incubated with secondary Alexa488-conjugated antibody 1:250 for 1 h at room temperature. Donkey Alexa488-conjugated IgG (Jackson ImmunoResearch Laboratories Inc., West Grove, PA) cat # 711-545-152 secondary antibody was used to detect rabbit primary antibody. After washing with PBS, the cells were incubated with Hoechst 33258 nucleic acid stain (1μg/mL) for 10 min. Finally, the coverslips were washed with PBS and mounted on glass slides with mounting medium. Images were acquired using a spectral laser scanning confocal Zeiss LSM800 fluorescent microscope (63X apochromatic, 1.4 NA objective) using dual excitation (493 nm for Alexa488 and 405 nm for Hoechst). Controls prepared by omission of primary antibody did not show any Alexa488 fluorescence under the above conditions.

### cAMP Radiobinding Protein Assay

For cAMP studies, HEK293T transfected cells were plated at 2x10^5^cells/cm^2^ in 48 wells plates. Two replicates per condition were performed in each independent experiment. Cardiomyocytes were maintained in 12 wells plate at an approximate density of 1.25x10^5^cells/cm^2^. Three replicates per condition were performed in each independent experiment.

For time-course cAMP accumulation experiments, cells were incubated 3 min in basal media supplemented with 1mM IBMX and exposed for different periods of time to 10µM BRL37344.

For response assays, cells were incubated 3 min in basal culture medium supplemented with 1mM IBMX at 37°C, followed by exposure to different concentrations of BRL37344 or forskolin for 30 min.

For β3AR blockade assays, cells were incubated 3 min in basal media supplemented with 1mM IBMX and 10µM L748337, and then exposed for 30 min to 1µM and 10µM BRL37344.

For desensitization assays, cells were pre-treated with 10µM BRL37344 in the absence of IBMX for different periods of time as shown in the figures. Cells were thoroughly washed and resuspended in fresh medium containing 1mM IBMX, incubated for 3 min, and exposed to 10µM BRL37344 for 30 min to determine whether the system was able to generate a cAMP response.

In all experiments, the reaction was stopped by ethanol addition followed by centrifugation at 3,000g for 5 min. The ethanol phase was then dried, and the residue resuspended in 50mM Tris-HCl pH 7.4, 0.1% BSA. cAMP content was determined by competition of [^3^H]cAMP for PKA, as previously described (Davio et al., 1995).

### FRET Time-Course of cAMP Intracellular Levels With Flex Station 3

FRET time-course of cAMP intracellular levels was measured as previously described (Carozzo et al., 2019). Briefly, HEKT Epac-S^H187^ transfected with pCDNA3.1 HA-β3-adrenergic receptor alone or with pCEFL GRK2 pC were seeded in 96-well plates at a density of 1 x 10^5^ cells/well. Before starting each experiment, cells were washed with NaCl 0.9% twice and 100 μl of FluoroBrite DMEM (Thermofisher) was added to each well before placing the plate in a FlexStation^®^ 3 at 37°C. In order to determine intracellular-cAMP (i-cAMP) response, we measured the baseline fluorescence signal detected at 475 nm (donor) and 530 nm (FRET) emission with excitation at 430 nm. Using the on-board pipettor, we added 50 μl of 3X BRL37344 after 40 s and then monitored the signal every 20 s for a total of 600 s. Different concentrations of BRL37344 were assayed for each transfection. FRET and donor intensities were measured for each time point. FRET/donor ratio was calculated and normalized to basal levels -before stimulation- (R/R_0_) for each time point. An AUC value of 10-minute R/R_0_ i-cAMP response was calculated for each replicate. Concentration-response curves were constructed plotting AUC values of 10-minute R/R_0_ i-cAMP response vs log [BRL37344].

### Radioligand Binding Assay

For binding assays, HEK293T cells transfected with pCDNA3.1 empty vector or pCDNA3.1 HA-β3-adrenergic receptor were seeded in 48 well plates at 7x10^4^ cells/well. The number of binding sites was evaluated by radioligand binding assay using [^3^H]-GCP12177 (30Ci/mmol) in the presence or absence of 8µM L748337 at 4°C. Binding in whole cells was performed at 4°C to avoid ligand internalization, as previously described (Tubio et al., 2010). The incubation was stopped by rapid washing with ice-cold physiologic solution (0.9% NaCl). After three washes, the bound fraction was collected in 200μl of ethanol, and Optiphase HiSafe3 scintillation cocktail was added to each fraction for counting in a HIDEX 300 SL counter.

To evaluate possible receptor internalization, transfected HEK293T cells were incubated 1 h with 10µM BRL37344 at 37°C, and after washing, the number of receptor sites was analyzed by radioligand binding assay.

### Quantitative RT-PCR

Total RNA was extracted from cardiomyocytes using Quick-Zol reagent (Kalium Technologies, Buenos Aires, Argentina) and reverse-transcribed using the High Capacity cDNA Reverse Transcription kit (Applied Biosystems, CA) following the manufacturer’s instructions. Quantitative RT-PCR (qPCR) was performed in triplicate using HOT FIREPol EvaGreen qPCR Mix Plus (Solis Biodyne, Tartu, Estonia) and a Rotor Gene Q detection system (Qiagen, Hilden, Germany), cycling parameters were as follows: 15 min at 95°C and 45 cycles of 30 s at 94°C, 30 s at 60°C, and 30 s at 72°C. The relative β3AR mRNA quantification was performed with the comparative ΔΔCt method using β-actin as the housekeeping gene.

### Co-Immunoprecipitation

HEK293T cells plated in 100mm dishes were co-transfected with β3AR, HA-Gαs and wild type GRK2 as indicated. 48 h after transfection cells were starved for 1 h, washed with PBS and incubated for 1 h at 37°C in PBS with 10µM BRL37344. Cross-linking was done in intact cells by replacement of stimuli by 3ml of PBS with 2.5 mM dithiobis(succinimidyl propionate) (DSP) (Pierce) with 10µM BRL37344 and incubation for 30 min at 25°C. DSP was washed with PBS and cells were solubilized in 1ml of radioimmune precipitation assay buffer (1% Nonidet P-40, 0.5% sodium deoxycholate, 0.1% SDS, 50mM Tris, pH 7.4, 100mM NaCl, 2mM EDTA, 50mM NaF, 1mM phenylmethylsulfonyl, 5µM aprotinin, 10µM leupeptin, 5µM pepstatin, 1mM sodium vanadate) at 4°C for 30 min, and immunoprecipitation of GRK2 was done by 2 h incubation with the specific anti-GRK2 antibody at 4°C. Immunocomplexes were recovered by overnight incubation with Protein A/G Plus-Agarose (Santa Cruz Biotechnology) and washed 4 times with ice-cold immunoprecipitation buffer. Separation of immune complexes and cleavage of the cross-linker was done for 90 min at 37°C in Laemmli buffer. Immunoprecipitated proteins were resolved by SDS-PAGE and transferred to nitrocellulose membranes. HA-Gαs and GRK2 were detected with a rabbit anti-HA antibody, rabbit anti-Gαs antibody or rabbit anti-GRK2 antibody as described below.

### Data and Statistical Analysis

Numbers (n) for all experiments are provided in corresponding figures legends and refer to independent measurements. In general, n=5 was used for concluding about mechanisms of action and n=3 was used for setting working conditions, characterizing our models or when different approaches of the same event (orthogonal assays) were followed. Data are presented as mean ± standard deviation (SD). Fittings of sigmoidal concentration-response, desensitization and binding saturation assay; and comparison of best fit values according to extra-sum of squares F test were performed with GraphPad Prism 6.00 for Windows, GraphPad Software (San Diego, CA). In radioligand binding assays specific binding was calculated by subtraction of nonspecific binding from total binding. For western blot data analysis, films were scanned and quantified using ImageJ software from National Institutes of Health (NIH) for the densitometry analysis of bands. For quantification, the background value of the scanned gel was subtracted, and the relative abundance was achieved by relativizing protein content to β-tubulin. Relative abundance was then normalized respect to control group.

Statistical analysis was carried out by one or two-sided Students’ t-test, one or two-way-ANOVA followed by Bonferroni or Dunnett post-test as indicated. Post hoc tests were run only if overall statistically significant diﬀerence between means were obtained. Statistical significance was accepted when P < 0.05. Statistics were performed using GraphPad 6.00 for Windows, GraphPad Software (San Diego, CA).

### Materials

Cell culture medium, antibiotics, isobutyl methylxanthine (IBMX), cAMP, bovine serum albumin (BSA), BRL37344, forskolin, L748337, amthamine, bromodeoxyuridine, holo-transferrin (human), insulin (bovine pancreas) pertussis toxin, and protease inhibitors were obtained from Sigma Chemical Company (St. Louis, MO). Dithiobis(succinimidyl propionate) (DSP) was from Pierce (Rockford, USA). FluoroBrite DMEM from Gibco was purchased from TermoFisher Scientific (New York, USA). [^3^H]cAMP, [^3^H](-)-CGP-12177 and Optiphase HiSafe3 scintillation cocktail were purchased from Perkin Elmer Life Sciences (Boston, MA). Foetal bovine serum was from Natocor (Córdoba, Argentina). Collagenase (type IV) was obtained from Worthington, (Lakewood, NJ). Other chemicals used were of analytical grade and obtained from standard sources.

Protein A/G Plus-Agarose (catalogue#SC-2003/Lot#J0118/Santa Cruz Biotechnology, CA)

The following primary antibodies were purchased from Santa Cruz Biotechnology, CA:

Anti-HA antibody (IgG from rabbit/catalogue#SC-805/Lot#K1915/final dilution: 1/500 for WB and 1/50 for immunofluorescence)
Anti-β-tubulin antibody (IgG from rabbit/catalogue#SC-9104/Lot#F1210/final dilution: 1/500)
Anti-GRK2 antibody (IgG from rabbit/catalogue#SC-562/Lot#F1610/final dilution: 1/1000)
Anti-GRK5 antibody (IgG from rabbit/catalogue#SC-565/Lot#D1210/final dilution: 1/400)
Anti-Gαs antibody (IgG from rabbit/catalogue#SC-383/Lot#L309/final dilution: 1/500)

The following secondary antibodies were purchased from Vector Laboratories, CA:

Anti-rabbit antibody (IgG from goat/catalogue#PI1000/Lot#X0126/final dilution: 1/4000)
Anti-mouse antibody (IgG from horse/catalogue#PI2000/Lot#W0218/final dilution: 1/2000)
Anti-rabbit antibody Alexa488-conjugated (IgG from donkey/cat # 711-545-152) was from Jackson ImmunoResearch Laboratories Inc., West Grove, PA)

Primers listed below were obtained from Genbiotech Oligos, BA:

β3AR_rat_FW: 5´-AACTCTGCCTTCAACCCGCTCAT-3´
β3AR_rat_RV:5´-TTCATGTGGGAAATGGACGCTCAC-3´
β-actin_rat_FW: 5´-CAGGGTGTGATGGTGGGTATGG -3´
β-actin_rat_RV: 5´-AGTTGGTGACAATGCCGTGTTC-3´

## Results

### Characterization of Human β3AR Response to BRL37344 in HEK293T Transfected Cells

**Figure 1:**
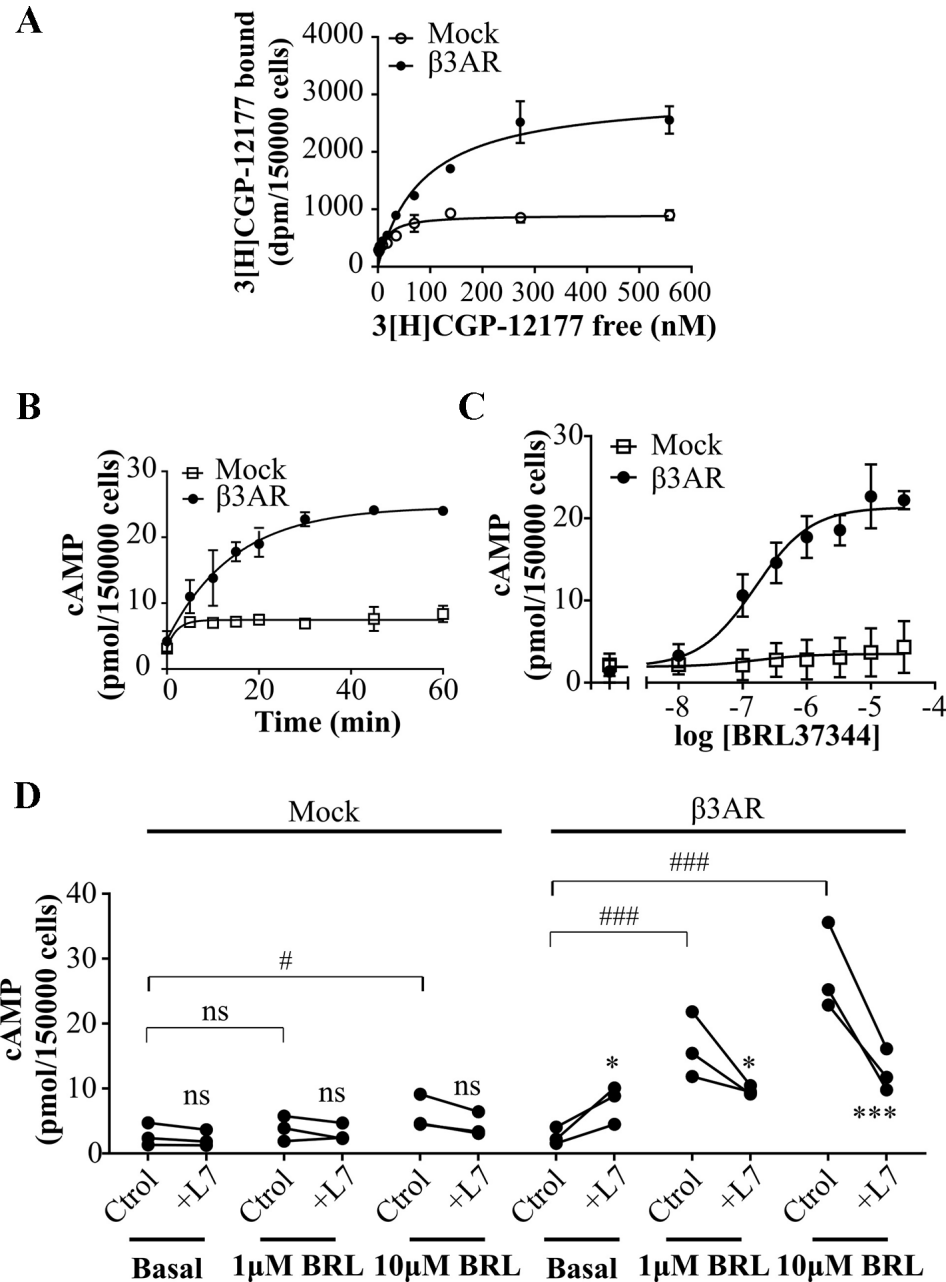
Characterization of cAMP response to β3AR stimulation. **(A)** HEK293T cells transfected with Mock or β3AR were incubated with different concentrations of ^3^[H]-CGP12177 as stated in *Materials and Methods*. Data represent mean ± SD (n=3). Best-fit values of the parameters are in the main text. Comparison of Kd and Bmax from both binding curves were done by extra sum of squares F-test. **(B)** HEK293T Mock or β3AR transfected cells were treated during different time periods with 10µM BRL37344, in presence of IBMX. Data represent mean ± SD (n=3). **(C)** HEK293T Mock or β3AR transfected cells were pre-treated with IBMX for 3 min and stimulated with indicated concentrations of BRL37344 for 30 min. Data represent mean ± SD (n=3). Best-fit values of the parameters are in the main text. **(D)** Effect of 10µM L748337 (L7) over basal or stimulated cAMP levels in response to 1μM or 10µM BRL37344 (BRL) in HEK293T Mock or β3AR transfected cells. Each data point represents a single experiment and data in absence or presence of L7 from the same experimental day are connected by lines (n=3). Data were analyzed by two-way ANOVA followed by Bonferroni post-test. ns, not significant; *P < 0.05; ***P < 0.001 respect to Ctrol. ns, not significant; ^#^P < 0.05; ^###^P < 0.001 as indicated. cAMP accumulation was quantified as described under *Materials and Methods*.

We initiated the study of the desensitization mechanisms of β3AR in a heterologous expression model, β3AR transfected HEK293T cells. In HEK293T cells transiently transfected with Mock or pCDNA3.1HA-β3AR (HEK293T-β3AR cells) we evaluated receptor expression by binding experiments using [^3^H]CGP-12177 a ligand for β1/2/3ARs commonly used as a general β blocker (Vrydag and Michel, 2007; Suarez et al., 2014; Gherbi et al., 2015; Littmann et al., 2015). We found a three-fold increase in [^3^H]CGP-12177 binding sites in HEK293T-β3AR (3024 ± 172) respect to Mock transfected cells (902 ± 50) (**Figure 1A**) and a right shift in Kd from 12.8 ± 3.2 to 85.9 ± 14.3. These results indicate the heterologous overexpression of the transfected β3AR in HEK293T cells.

In a first attempt to time-resolve cAMP response of β3AR, HEK293T-β3AR cells were stimulated during different time periods with the β3AR agonist, BRL37344 (Alexander et al., 2013) in presence of IBMX (phosphodiesterase inhibitor), and cAMP accumulation was determined. Maximal cAMP levels were reached after 30 min treatment (**Figure 1B**). Considering this, concentration-response experiments were done by stimulating cells for 30 min with BRL37344. In Mock transfected HEK293T cells, Rmax to BRL37344 was 4.5 ± 1.7 pmol/150,000 cells. As expected, a significantly higher production of cAMP was observed in HEK293T-β3AR cells, where Rmax was 21.4 ± 0.5 pmol/150,000 cells (P < 0.05) (**Figure 1C**). These results indicate that in our system, endogenously expressed receptors and transfected HA-β3AR behave similar, with both concentration-response curves fitting to a model showing a common pEC50 value of 6.79 ± 0.17, whereas the maximal response was approximately 5-fold higher in HEK293T-β3AR cells. Pre-treatment with L748337, a specific β3AR ligand commonly used as β3AR blocker (Sato et al., 2008; Alexander et al., 2013) although led to a low increase in cAMP basal levels, completely impeded cAMP increased in response to BRL37344 (**Figure 1D**), confirming that in our system, the observed cAMP response to BRL37344 is specifically due to β3AR stimulus.

### Short-Term Desensitization of Human β3AR in HEK293T Transfected Cells

**Figure 2:**
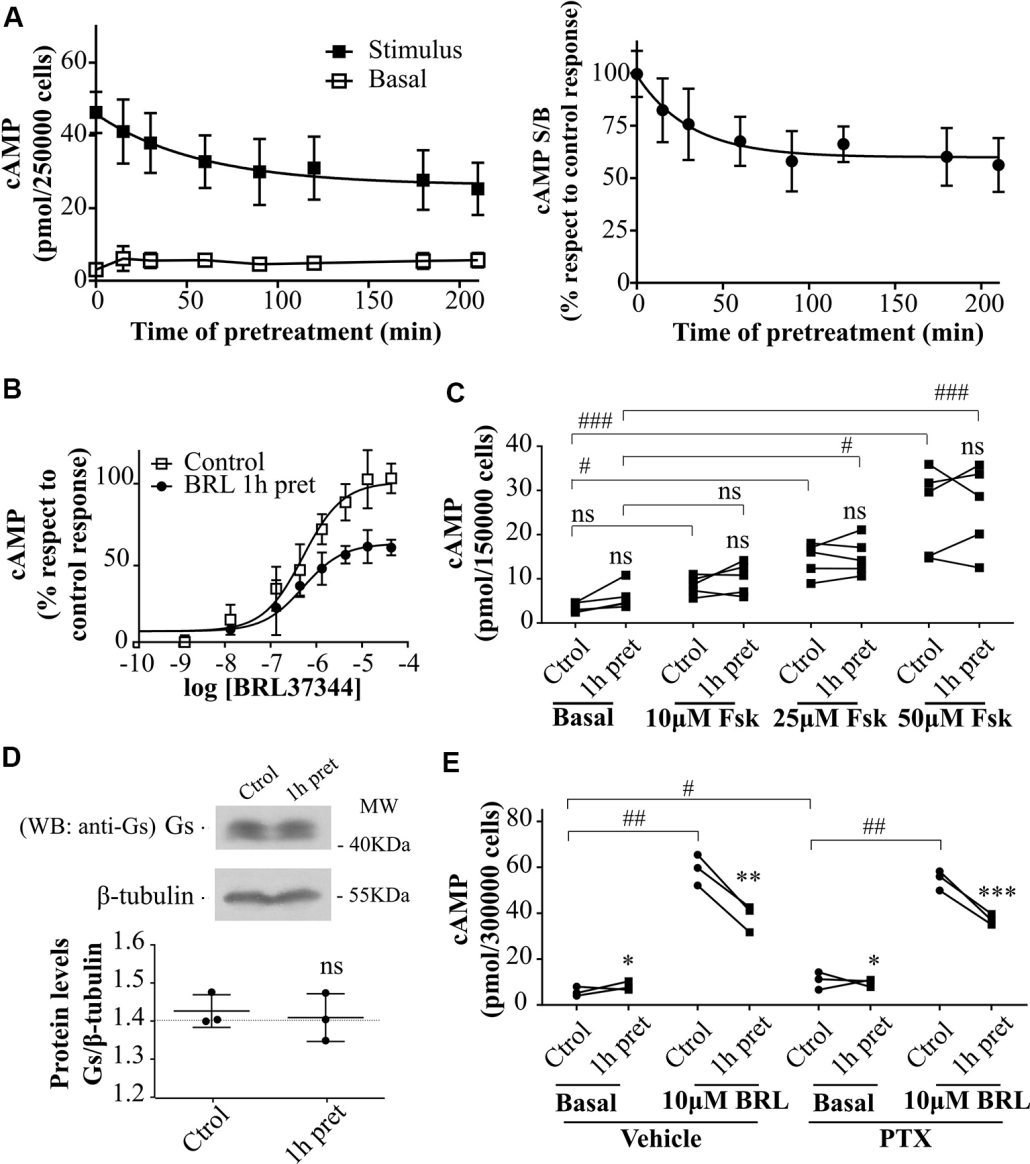
Desensitization of β3AR cAMP response. **(A)** HEK293T-β3AR cells were pre-treated during different time-periods with 10µM BRL37344. After that, cells were washed, incubated with IBMX for 3 min and challenged (stimulated response) or not (basal response) with 10µM BRL37344 for 30 min. cAMP levels are expressed as picomols over 250000 cells (left panel) or as the ratio of stimulated over basal cAMP (S/B) respect to control (without pre-treatment) which is considered as 100% (right panel). Data represent mean ± SD (n=5). Best-fit values of the parameters are in the main text. **(B)** HEK293T-β3AR cells were pre-treated with 10µM BRL37344 for 1h or not and stimulated with different concentrations of BRL37344. Data represent mean ± SD (n=5). Best-fit values of the parameters are in the main text. **(C)** HEK293T-β3AR cells were pre-treated with 10µM BRL37344 for 1h or not and stimulated with different concentrations of forskolin (Fsk) for 30 min. Each data point represents the mean of a single experiment and cAMP response in untreated or pre-treated cells from the same experimental day are connected by lines (n=5). ns indicates not significant differences respect to Ctrol analyzed by two-way ANOVA followed by Bonferroni post-test. ns, not significant; ^#^P < 0.05; ^###^P < 0.001 as indicated. **(D)** HEK293T-β3AR cells were pre-treated with 10µM BRL37344 for 1h or not, lysed, resolved by 12% SDS-PAGE and immunoblotted using anti-Gαs antibody. A representative image is showed. Densitometry analysis was performed with ImageJ as indicated in *Materials and Methods* and analyzed by student t-test comparing Gαs levels in 1 h pre-treated vs control cells (n=3). **(E)** Basal or 10µM BRL37344 stimulated cAMP levels were determined in control or desensitized HEK293T-β3AR cells treated with PTX 100ng/mL or vehicle for 3h. Data represent mean ± SD. Each data point represents the mean of a single experiment and cAMP response in untreated or BRL37344 pre-treated cells from the same experimental day are connected by lines (n=3). Data were analyzed by two-way ANOVA followed by Bonferroni post-test. *P < 0.05; **P < 0.01; ***P < 0.001 respect to Ctrol. ^#^P < 0.05; ^##^P < 0.01 as indicated. cAMP production was quantified as described under *Materials and Methods*.

To evaluate the effect of agonist pre-treatment on β3AR responsiveness, HEK293T-β3AR cells were pre-incubated with 10µM BRL37344 for a period ranging from 0 to 210 min. The pre-exposure of HEK293T-β3AR cells to BRL37344 although slightly increased basal levels notably diminished cAMP production when cells were re-challenged with the same agonist (**Figure 2A** left panel). Plateau response induced by BRL37344 was almost 59 ± 13% (**Figure 2A** right panel) of the control response of 45.9 ± 2.7 pmol/250,000 cells, and half maximal desensitization was observed at 27 min (**Figure 2A**). These results were confirmed by concentration-response assays in control or 1h pre-treated HEK293T-β3AR cells. Pre-treatment of the cells with 10µM BRL37344 significantly diminished concentration dependent response to BRL37344 with Rmax varying from 21.5 ± 0.9pmol/150,000 cells to 16.0 ± 0.7pmol/150,000 cells (P < 0.05) (**Figure 2B**). Interestingly, cAMP response to forskolin, a direct activator of adenylyl cyclase, was not decreased by pre-treatment of cells (**Figure 2C**). This result indicates that the events involved in the attenuation of β3AR response takes place upstream AC activation. A reduction in Gαs protein levels upon 24 h treatment with isoprenaline was described by other authors in HEK293 cells (Michel-Reher and Michel, 2013). However, 1 h treatment with BRL37344 did not reduce immunoreactivity of Gαs in HEK293T-β3AR (**Figure 2D**). Since β3AR can also couple to Gαi (Chaudhry and Granneman, 1994; Pott et al., 2006) we evaluated whether changes in receptor coupling to G protein could be responsible for the diminished cAMP response to BRL37344 observed after pre-exposure to the agonist. Inhibition of Gαi protein by pre-treatment of HEK293T-β3AR cells with pertussis toxin (PTX) although increase basal cAMP levels did not prevent desensitization of cAMP response after pre-exposure to BRL37344 (**Figure 2E**).

### GRKs Involvement in Human β3AR Signaling Regulation

**Figure 3:**
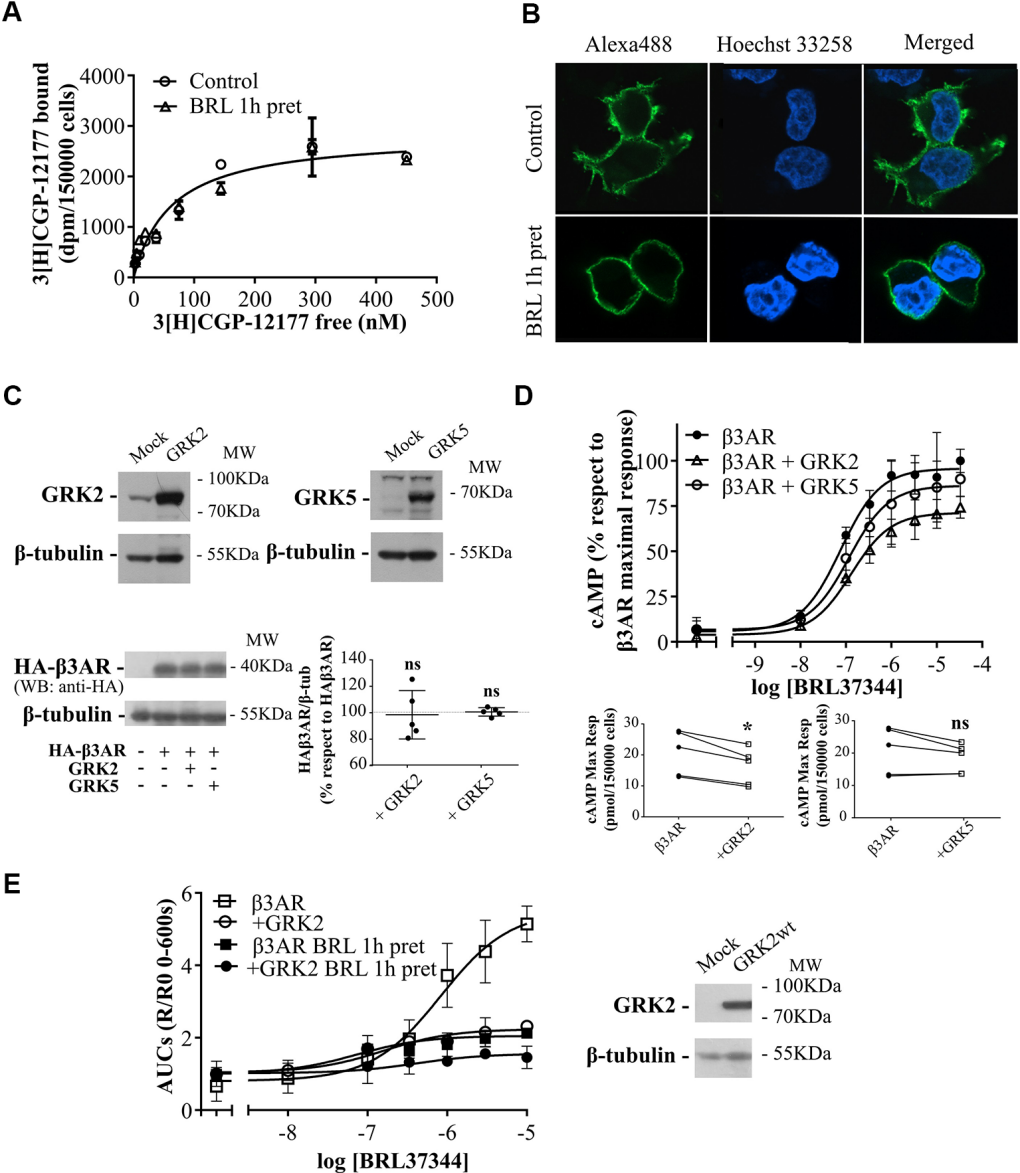
Mechanisms of regulation of β3AR cAMP response. **(A)** HEK293T-β3AR cells were pre-treated with 10µM BRL37344 for 1h or not, washed and incubated with different concentrations of ^3^[H]-CGP12177 as stated in *Materials and Methods*. Data represent mean ± SD (n=3). Best-fit values of the parameters are in the main text. Comparisons of Bmax from both binding curves were done by extra sum of squares F-test. **(B)** HEK293T cells transfected with HAβ3AR were pre-treated with 10µM BRL37344 for 1h or not, washed and fixed for immunocytochemical staining as described under *Materials and Methods*. Images of fluorescence signal corresponding to secondary Alexa488-conjugated antibody, nuclear Hoechst 33258 staining and merged images are shown. The immunostaining obtained with the anti-HA tag antibody indicated expression and location of HAβ3AR in HEK293T cells after transfection. Images are representative of 3 independent experiments. **(C)** HEK293T-β3AR cells co-transfected with GRK2 or GRK5 were lysed, resolved by 12% SDS-PAGE and immunoblotted using anti-GRK2, anti-GRK5, anti-HA or anti-β tubulin antibodies. A representative image is showed. Densitometry analysis was performed with ImageJ as indicated in *Materials and Methods* and analyzed by student t-test comparing HA-β3AR levels in GRK2 or GRK5 co-transfected cells vs HA-β3AR without GRKs which was considered as 100%. Results are mean ± SD (n=5). **(D)** HEK293T-β3AR cells co-transfected with GRK2 or GRK5, were pre-treated with IBMX for 3 min and stimulated with different concentrations of BRL37344 for 30 min. Upper panel: results are expressed as % respect to maximal response of control (β3AR without GRKs), data represent mean ± SD (n=5). Lower panels: Raw data of the maximal cAMP levels from each individual experiment are shown. Data were analyzed by paired student t-test. ns, not significant; *P < 0.05 respect to β3AR. cAMP production was quantified as described under *Materials and Methods*. **(E)** HEKT Epac-S^H187^ transiently co-transfected with β3AR and GRK2 wild type were pre-treated during 1 h with either 10µM BRL37344 or vehicle. After that, cells were washed and challenged with different concentrations of BRL37344. Concentration-response curves were constructed with the AUC values of 10-minute R/R0 i-cAMP response of time course of FRET changes determined in FlexStation^®^ 3 at 37°C as described in *Materials and Methods* (left). HEKT Epac-S^H187^ co-transfected with β3AR and GRK2 wild type, were lysed, resolved by SDS-PAGE 12% and immunoblotted using anti GRK2 or anti-β tubulin antibodies as indicated. Image showed is representative of three independent experiments (right).

To determine whether the decrease in cAMP response in pre-treated cells was due to a reduction in β3AR sites we carried out binding experiments using [^3^H]CGP-12177. Results demonstrated that 1h pre-treatment with 10µM BRL37344 did not alter the density of [^3^H]CGP-12177 binding sites in HEK293T-β3AR cell membranes, showing a global Bmax of 2840 ± 147 sites in control and pre-treated cells (**Figure 3A**). In immunofluorescence assays we observed by confocal microscopy of HEK293T-β3AR that HA-β3AR was present at the plasma membrane. In cells treated for 1h with 10µM BRL37344, no change in the subcellular distribution of β3AR was observed (**Figure 3B**). These results indicate that pre-treatment with BRL37344 does not lead to internalization of β3AR sites.

Considering that GRKs are determinants of GPCR signaling and taking into account that GRK2 and GRK5 are the most abundant GRKs in the cardiovascular system (Harris et al., 2008; Dorn, 2009) where a cardioprotective role was conferred to β3AR agonism (Dessy and Balligand, 2010; Niu et al., 2012; Cannavo and Koch, 2017) we investigated whether these proteins were involved in β3AR desensitization. Concentration–response experiments were carried out after 30 min stimulation with increasing concentrations of BRL37344 in HEK293T-β3AR cells co-transfected alternatively with GRK2 or GRK5 constructs. We observed that although β3AR overexpression is not altered when co-transfected with either GRK2 or GRK5 (**Figure 3C**) the cAMP response of the receptor varied depending on the subtype of GRK overexpressed (**Figure 3D**). GRK2 overexpression but not GRK5 significantly decreased concentration dependent response to BRL37344 in a 27 ± 2% respect to HEK293T-β3AR control cell (P < 0.05) (**Figure 3D**). Since cAMP accumulation studies in presence of IBMX have been recently questioned based on the possibility that accumulating cAMP might leave the cells and act on adenosine receptors lowering intracellular cAMP levels (Okeke et al., 2019), we confirmed our studies using FRET based determinations to quantified real-time intracellular cAMP (i-cAMP) levels without IBMX (**Figure 3E**). Using this methodology, we observed a more pronounced effect of GRK2 overexpression and/or pre-exposure to the agonist over cAMP production in response to β3AR stimulation.

### RGS-Mediated Regulation of Human β3AR in HEK293T Transfected Cells

**Figure 4:**
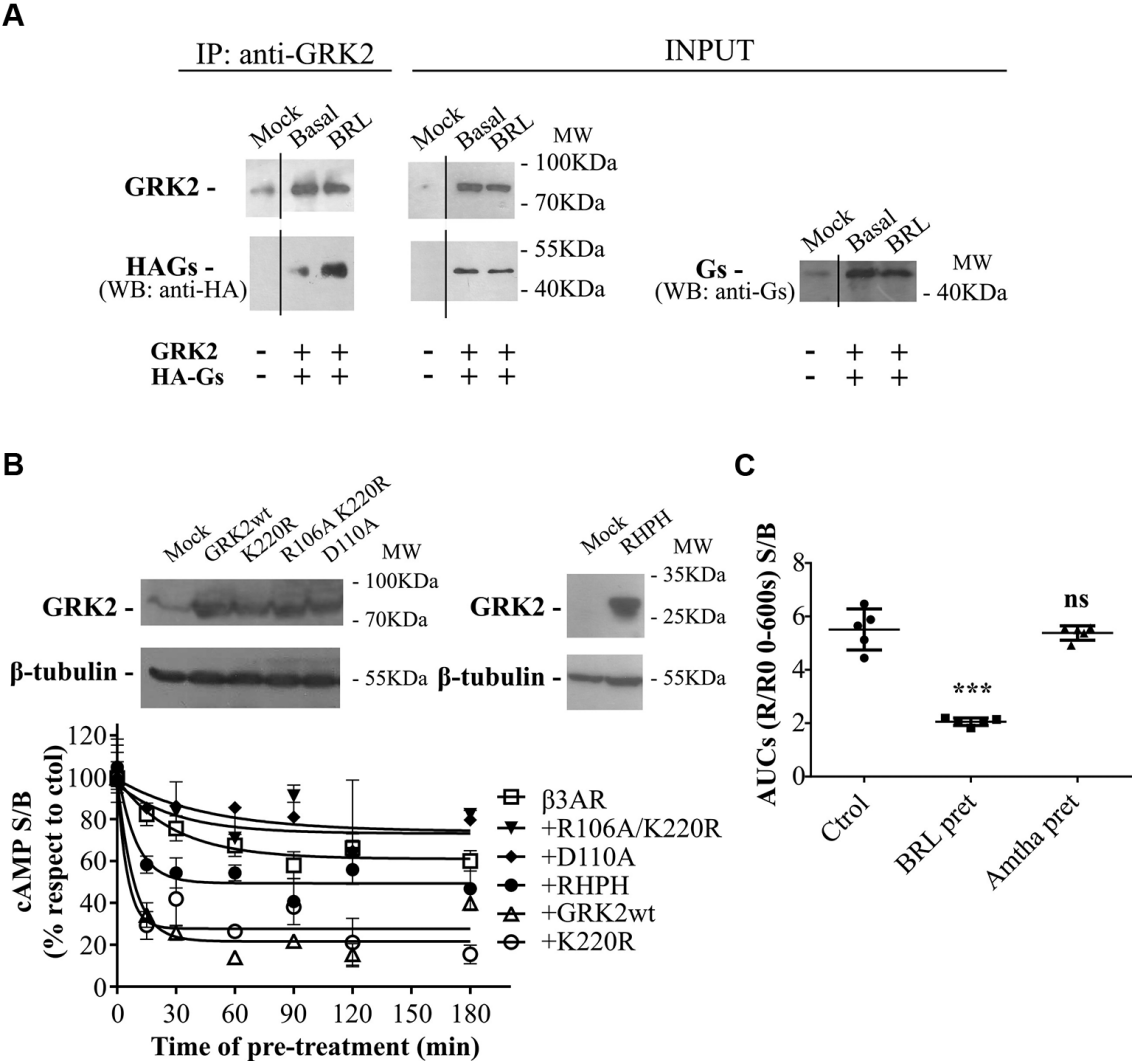
GRK2-mediated desensitization of human β3AR. **(A)** Co-immunoprecipitation of Gαs and GRK2 is shown. HEK293T cells co-transfected with β3AR, HA-tagged Gαs and GRK2 or Mock were incubated for 10 min with 10µM BRL37344, and cross-linking with 2.5mM dithiobis(succinimidyl propionate) was done. Cells were lysed, and GRK2 was immunoprecipitated (IP) using anti-GRK2 antibodies followed by A/GPlus agarose beads incubation. Co-precipitated Gαs was detected by western blot using anti-HA antibodies. Total Gαs was detected by western blot using anti-Ha or anti-Gαs antibodies. **(B)** HEK293T-β3AR cells co-transfected with GRK2 wild type, GRK2-K220R, GRK2-D110A, GRK2-R106A/K220R or RHPH(GRK2) were lysed, resolved by SDS-PAGE 12% and immunoblotted using anti-GRK2 or anti-β tubulin antibodies as indicated. Image showed is representative of three independent experiments (upper panel). Transfected HEK293T cells were pre-treated during different time periods with 10µM BRL37344. After that, cells were washed, incubated with IBMX for 3 min and challenged (stimulated response) or not (basal response) with 10µM BRL37344 for 30 min. cAMP production was quantified as described under *Materials and Methods* (lower panel). Data is shown as stimulated over basal response (S/B) normalized respect to control (without pre-treatment) considered as 100% and represent mean ± SD (n=3). Best-fit values of the parameters are in the main text. **(C)** HEKT Epac-S^H187^ transiently co-transfected with β3AR and H2R were pre-treated during 1 h with either 10µM BRL37344, 10µM amthamine or vehicle. After that, cells were washed and challenged with 10µM BRL37344. cAMP response was constructed with the AUC values of 10-minute R/R0 i-cAMP response of time course of FRET changes determined in FlexStation^®^ 3 at 37°C as described in *Materials and Methods*. Data is shown as stimulated over basal response (S/B) and analyzed by one-way ANOVA and Dunnett post-test. ns, not significant; ***P < 0.001 respect to Ctrol. Results are mean ± SD (n=5).

We have previously reported that the regulator of G-protein signaling (RGS) homology domain of GRK2 (RH) is sufficient to mediate the desensitization of a Gαs coupled GPCR, the histamine type 2 receptor (H2R) (Fernandez et al., 2011). Moreover, RH has been involved in phosphorylation independent signaling attenuation of several GPCRs (Sallese et al., 2000; Usui et al., 2000; Reiter et al., 2001; Dhami et al., 2002; Iwata et al., 2005; Namkung et al., 2009; Fernandez et al., 2011; Echeverria et al., 2019). When cells are stimulated with 10µM BRL37344 a direct interaction between wild type GRK2 and HA-Gαs is observed by co-immunoprecipitation assay (**Figure 4A**). Based on these observations and given that β3AR was described to be not susceptible to phosphorylation, we explored the influence of RH domain of GRK2 in the regulation of β3AR responsiveness.

Time-course desensitization experiments were performed through cAMP levels determinations in HEK293T-β3AR cells co-transfected with GRK2 wild type or dominant negative mutants for kinase (GRK2-K220R) or RH (GRK2-D110A) or both (GRK2-R106A/K220R). For this, cells were pre-treated with agonist during different periods of time and re-challenged with the same ligand. The expression of the different GRK2 variants was detected by western blot (**Figure 4B** upper panel). In HEK293T-β3AR cells overexpressing GRK2, pre-treatment with BRL37344 decreased cAMP response of β3AR to a re-challenge with the agonist in a more pronounce manner than in HEK293T-β3AR control cells (**Figure 4B** lower panel). While HEK293T-β3AR control cells reached a plateau of 61.03 ± 3.53% of the control response, cells overexpressing GRK2 presented a significantly lower plateau of 21.62 ± 3.34% of the control response (P < 0.01), showing an inhibition in the response to BRL37344. In the same way, overexpression of GRK2-K220R (kinase inactive) or the RH domain fused to the C-terminal PH domain of GRK2 (RHPH) also increased β3AR desensitization, leading to a residual response with a plateau of 27.70 ± 4.71% (P < 0.01) or 49.37 ± 3.39% (P < 0.05) of the control response respectively. On the contrary, overexpression of variants with mutated RH domain GRK2-D110A or GRK2-R106A/K220R although tended to diminish β3AR desensitization 74.06 ± 15.86% and 73.16 ± 9.21 showed no significant differences in the residual response respect to the control cells (**Figure 4B** lower panel).

These results point to the RH domain of GRK2 as responsible for β3AR desensitization and indicate that a functional RH domain of GRK2 is necessary to achieve complete β3AR desensitization. Based on that, we next evaluated whether prolonged activation of other Gαs coupled GPCR as the H2R may also lead to β3AR desensitization. Pre-treatment of cells for 1h with H2R agonist amthamine 10µM although led to H2R desensitization (data not shown) did not modify β3AR cAMP response to BRL37344 (**Figure 4C**).

### RH-Mediated Regulation of Endogenous β3AR in Neonatal Rat Cardiomyocytes

**Figure 5:**
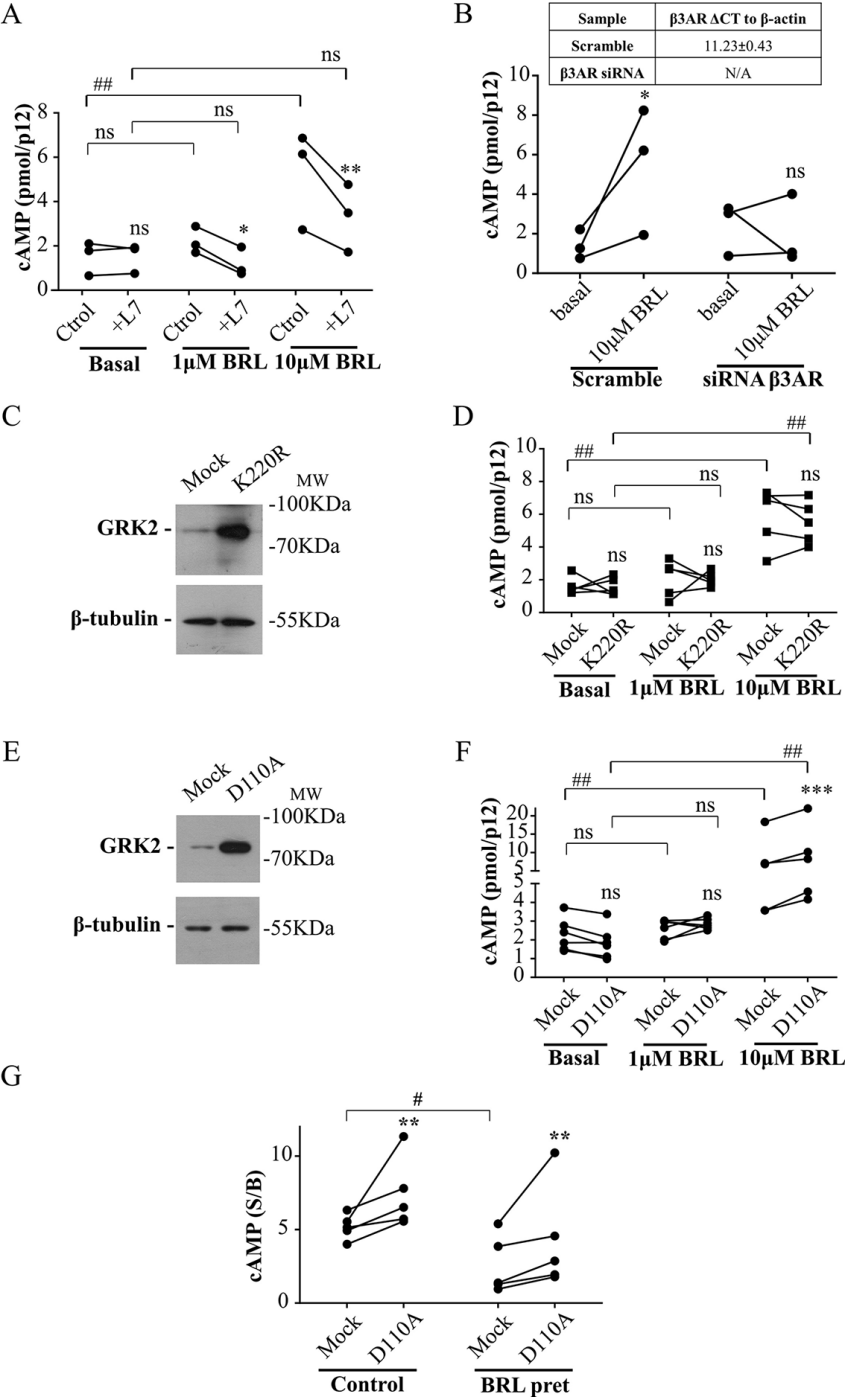
Involvement of RH domain of GRK2 in the regulation of β3AR response in cardiomyocytes. **(A)** Neonatal rat cardiomyocytes cells were incubated with IBMX for 3 min, treated with different concentrations of BRL37344 in the presence or not of 10µM of β3AR antagonist L748337 as indicated. Each data point represents the mean of a single experiment and cAMP response in control or L748337 pre-incubated cells from the same experimental day are connected by lines (n=3). Data were analyzed by two-way ANOVA followed by Bonferroni post-test. ns, not significant; *P < 0.05; **P < 0.01 respect to Ctrol. ns, not significant; ##P < 0.01 as indicated. **(B)** Neonatal rat cardiomyocytes transfected with Mock or siRNA targeting endogenous β3AR were pre-treated with IBMX for 3 min and stimulated with 10µM BRL37344 for 30 min. Each data point represents the mean of a single experiment and cAMP levels in basal o BRL stimulated cells from the same experimental day are connected by lines (n=3). Data were analyzed by two-way ANOVA followed by Bonferroni post-test. ns not significant, *P < 0.05 respect to basal. **(C, E)** Neonatal cardiomyocytes cells transfected with GRK2-K220R or GRK2-D110A were lysed, resolved by 12% SDS-PAGE and immunoblotted using anti-GRK2 or anti-β tubulin antibodies. Image showed is representative of three independent experiments. **(D, F)** Cardiomyocytes transfected with Mock, GRK2-K220R **(D)** or GRK2-D110A **(F)** were pre-treated with IBMX for 3 min and stimulated with BRL37344 for 30 min at indicated concentrations. Each data point represents the mean of a single experiment and cAMP response in Mock and K220R **(D)** or Mock and D110A **(F)** transfected cells from the same experimental day are connected by lines (n=5). Data were analyzed by two-way ANOVA followed by Bonferroni post-test. ns, not significant; ***P < 0.001 respect to Mock. ns, not significant; ##P < 0.01 as indicated. **(G)** Cardiomyocytes transfected with Mock or GRK2-D110A were pre-exposed to 1µM BRL3734 for 30 min, washed, incubated with IBMX for 3 min and stimulated with 10µM BRL37344 for 30 min. Each data point represents the mean of a single experiment and cAMP response in Mock and GRK2-D110A transfected cells from the same experimental day are connected by lines (n=5). Data is shown as stimulated over basal response (S/B). Data were analyzed by two-way ANOVA followed by Bonferroni post-test. **P < 0.01 respect to Mock. #P < 0.05 as indicated. cAMP production was quantified as described under *Materials and Methods*.

Cardiac β3AR over-expression and signaling proved to be a cardioprotective event in patients with heart failure (Niu et al., 2012). Based on that, we aimed to evaluate whether interruption of the desensitization mediated by the RH domain of GRK2 allows an increased response of β3AR in a primary culture of rat cardiomyocytes. Since there is a minority cardiac expression of β3AR respect to that of β1AR or β2AR (Pott et al., 2006), we first analyzed the specificity of the cAMP response to BRL37344. In primary cultured rat cardiomyocytes, while 1µM BRL37344 did not significantly increase cAMP levels, stimulation with 10µM BRL37344 led to a significant cAMP response that was inhibited by L748337 (**Figure 5A**). Cardiomyocytes transfected with siRNA for β3AR, that led to knockdown of receptor levels to undetectable amounts by qPCR (**Figure 5B** table), showed no cAMP production after stimuli with 10µM BRL37344, confirming the specificity of the evaluated response (**Figure 5B**).

Transfection of cardiomyocytes with GRK2-K220R mutant lacking kinase activity but with intact RH domain (**Figure 5C**), did not modify cAMP response to BRL37344 (**Figure 5D**) while transfection with GRK2-D110A (**Figure 5E**) significantly augmented cAMP response to 10µM BRL37344 (**Figure 5F**). In the same way, 30 min pre-treatment of cardiomyocytes with 1µM BRL37344, significantly diminished the capacity of response to BRL37344 (**Figure 5G**) indicating β3AR desensitization. However, in desensitized myocytes cAMP response to BRL37344 was higher in cells transfected with GRK2-D110A than in Mock transfected cells (**Figure 5G**). These results indicate that inhibition of RGS activity of GRK2 allows a higher β3AR signaling and points out RH domain as a relevant regulator of β3AR response in the cardiac context.

## Discussion

For a long time, β3AR has been thought to be insensitive to PKA or GRK mediated desensitization due to the lack of potential phosphorylation sites in its third intracellular loop and c-terminal tail that are indeed present in β1AR and β2AR variants. This observation was confirmed by results obtained using chimeric β2/3-AR (Hausdorff et al., 1990; Liggett et al., 1993; Jockers et al., 1996). However, experiments carried out in heterologous expression systems allowed to describe that long-term stimulation of human β3AR leads to attenuation of receptor response in HEK293 transfected cells (Vrydag et al., 2009; Michel-Reher and Michel, 2013; Okeke et al., 2019). Results presented in this work show that human β3AR heterologously expressed in HEK293T cells is sensitive to short-term desensitization by a mechanism that takes place upstream AC activation and does not involve Gαs downregulation nor a switch in receptor coupling to Gαi. Instead, it proved to depend on the integrity of the RH domain of GRK2.

RGS family of proteins regulates GPCR signaling through two general mechanisms. One involves its GAP (GTPase-accelerating protein) activity, which favors Gα inactivation and the other relies on the ability of RGS domain to bind activated Gα protein subunit precluding the activation of the effector protein (e.g. AC) (Sterne-Marr et al., 2004). Considering that it has been demonstrated that GRK2 has weak GAP activity (Carman et al., 1999) and based on our findings where Gαs co-immunoprecipitates with GRK2 after β3AR stimulation, we hypothesize that the underlying mechanism of the observed β3AR short-term desensitization might be based on the effector antagonism mediated by the RH domain of GRK2. Hence, β3AR regulation in HEK293T transfected cells might be bimodal, including a fast inactivation of receptor response at the level of G protein by RH mediated blockade of Gαs signaling, followed by a long-term desensitization by adenylyl cyclase inactivation and Gαs downregulation (Michel-Reher and Michel, 2013). These results agree with previous observations indicating that the target in β3AR desensitization is not the receptor itself (expression and density) but the signaling cascade instead (Seifert, 2013). While long-term mechanisms might have a more profound and generalized effect over signaling of GPCRs coupled to Gαs, RH driven desensitization by GRK2 might be more specific since soluble GRK2 translocates to plasmatic membrane after receptor activation, affecting only the signaling of the stimulated GPCR. Consistently, in HEK293T cells transfected with H2R and β3AR desensitization of H2R did not attenuate β3AR response, even though both GPCRs share that they are desensitized by the RH domain of GRK2 (Fernandez et al., 2011). Also, the possibility of other members of RGS family taking place in β3AR signaling regulation should be considered and further studied.

Endogenously expressing β3AR models yielded heterogeneous results regarding receptor desensitization and in this context, transfected cells allowed us to identify underlying molecular mechanisms. However, HEK293T transfected cells may not be representative of tissues that endogenously express β3AR. Previous reports indicate that β3AR plays an important role in cardiomyopathies. Its expression levels were shown to be increased in human ischemic and dilated ventricles of failing hearts (Moniotte et al., 2001). This increment was accompanied by a more prevailing β3AR function over β1/2AR counterparts (Gauthier et al., 2000). Based on that, it has been suggested a promising therapeutic strategy to treat heart failure based on long-term β3AR stimulation that would protect against the adverse remodelling mainly mediated by β1AR response to the increased sympathetic activity (Engelhardt et al., 1999; Cannavo and Koch, 2017). In this context, GRK2 takes relevance, as chronic exposure to catecholamines is followed by its overexpression in the failing heart (Campanile and Iaccarino, 2009). Therefore, since pathological alteration of GRK2 levels and function might also be detrimental for β3AR cardioprotective function, proper identification and characterization of the mechanisms of β3AR desensitization is of paramount importance. Accordingly, we studied receptor response in a cardiac environment where physiological levels of β3AR are expressed. Since cardiac cell lines do not precisely depict the functional physiology of isolated ventricular cardiomyocytes, we employed primary cardiomyocytes culture as a representative model of cardiac signaling at the cellular level and evaluated the relevance of RGS-GRK2 mediated regulation on β3AR response. Although β3AR has been described to preferentially couple to Gαi in the human heart in native conditions, it has been proved to also couple to Gαs-AC-PKA in cardiac tissue of transgenic rodent model (Kohout et al., 2001) as well as in heterologous expression system and adipocytes (Soeder et al., 1999). Our results indicate that in rat cardiomyocytes, activation of endogenously expressed β3AR increases cAMP intracellular levels, and β3AR undergoes short-term desensitization. We also observed that over-expression of a dominant negative mutant of RH domain of GRK2, increases receptor responsiveness and attenuates desensitization, suggesting the involvement of this domain of GRK2 in the regulation of cardiac expressed β3AR.

Although these findings agree with the mechanism described in HEK293T cells some differences between models are observed and the mechanism described in HEK293T cells may not be extrapolated to cardiomyocytes. For example, it is noticeable that RGS dominant negative mutants are more effective in cardiomyocytes (**Figures 5D–G**) and overexpression of RH functional domain seems to have more radical effects in HEK293T cells (**Figure 4B**). These apparent discrepancies could be explained in terms of differences in the relative expression levels of endogenous GRK2 between the two models. On the other hand, no significant cAMP response to 1µM BRL37344 was observed in cardiomyocytes. However, the efficacy of a ligand is directly related to receptor density, and then, it is not surprising that a ligand proves to be more efficacious in an overexpression system than in a not transfected primary culture.

Overall, results presented here show the role of RH domain of GRK2 in the desensitization of β3AR, a GPCR that has been proposed as a therapeutic target for long term treatment of heart failure. Additionally, mirabegron has been licensed as a first-in-class β3AR agonist for the treatment of overactive bladder syndrome and has shown to be well tolerated and effective. In the same way, vibegron (Yoshida et al., 2018) is being studied for the same purpose. However, treatment with these ligands do not lead to resolution of the syndrome and long-term use of the agonist is also needed to improve bladder symptoms. Although mirabegron is used at nanomolar concentrations (Silva et al., 2017), the existence of receptor desensitization has not been rule out, and considering the results presented herein, further understanding and characterization of the mechanisms involved in β3AR desensitization would allow overcoming potential limitations that chronic use of agonists may bring in a long-term therapy of heart failure or overactive bladder syndrome.

## Data Availability Statement

The datasets generated for this study are available on request to the corresponding author.

## Ethics Statement

The animal study was reviewed and approved by the Animal Use and Care Committee from the Facultad de Farmacia y Bioquímica, Universidad de Buenos Aires.

## Author Contributions

Participated in research design: EE, MC, VB, FM, NF. Conducted experiments: EE, MC, VB, MS, SR, CD, NF. Contributed new reagents or analytic tools: AY, CD, CS. Performed data analysis: EE, FM, CD, NF. Wrote or contributed to the writing of the manuscript: EE, VB, AY, FM, CS, NF. All authors participated in revising the manuscript and approved final version.

## Funding

This work was supported by: Universidad de Buenos Aires (UBACyT 20020130100624BA); Agencia Nacional de Promoción Científica y Tecnológica (PICT 2013 N°2050, PICT 2015 N° 2443); and Consejo Nacional de Investigaciones Científicas y Técnicas (2013-2015 PIP 562).

## Conflict of Interest

The authors declare that the research was conducted in the absence of any commercial or financial relationships that could be construed as a potential conflict of interest.

## Abbreviations

AC, adenylyl cyclase; β3AR, β3-adrenergic receptor; FRET, Föster Energy Transfer; GAP, GTPase accelerating protein; GRK, G protein coupled receptor kinase; H2R, Histamine receptor type 2; PKA, cAMP dependent protein kinase; RGS, regulator of G protein signaling; RH; homologous to RGS; siRNA, small interference RNA.

**Chemical compounds studied in this article:** BRL37344 (PubChem CID: 9841972); L748337 (PubChem CID: 10028830); Forskolin (PubChem CID: 47936); [^3^H]CGP-12177 (PubChem CID: 101178785); Amthamine (Pubchem CID: 126688).
